# Development and Evaluation of a Next-Generation Digital PCR Diagnostic Assay for Ocular Chlamydia trachomatis Infections

**DOI:** 10.1128/JCM.00622-13

**Published:** 2013-07

**Authors:** Chrissy h. Roberts, Anna Last, Sandra Molina-Gonzalez, Eunice Cassama, Robert Butcher, Meno Nabicassa, Elizabeth McCarthy, Sarah E. Burr, David C. Mabey, Robin L. Bailey, Martin J. Holland

**Affiliations:** Clinical Research Department, London School of Hygiene and Tropical Medicine, London, United Kingdoma; Department of Immunology, London School of Hygiene and Tropical Medicine, London, United Kingdomb; Medical Research Council Unit, The Gambiac; Programa Nacional de Saude de Visao, Ministerio de Saude Publica, Bissau, Guinea Bissaud

## Abstract

Droplet digital PCR (ddPCR) is an emulsion PCR process that performs absolute quantitation of nucleic acids. We developed a ddPCR assay for Chlamydia trachomatis infections and found it to be accurate and precise. Using PCR mixtures containing plasmids engineered to include the PCR target sequences, we were able to quantify with a dynamic range between 0.07 and 3,160 targets/μl (*r*^2^ = 0.9927) with >95% confidence. Using 1,509 clinical conjunctival swab samples from a population in which trachoma is endemic in Guinea Bissau, we evaluated the specificity and sensitivity of the quantitative ddPCR assay in diagnosing ocular C. trachomatis infections by comparing the performances of ddPCR and the Roche Amplicor CT/NG test. We defined ddPCR tests as positive when we had ≥95% confidence in a nonzero estimate of target load. The sensitivity of ddPCR against Amplicor was 73.3% (95% confidence interval [CI], 67.9 to 78.7%), and specificity was 99.1% (95% CI, 98.6 to 99.6%). Negative and positive predictive values were 94.6% (95% CI, 93.4 to 95.8%) and 94.5% (95% CI, 91.3 to 97.7%), respectively. Based on Amplicor CT/NG testing, the estimated population prevalence of C. trachomatis ocular infection was ∼17.5%. Receiver-operator curve analysis was used to select critical cutoff values for use in clinical settings in which a balance between higher sensitivity and specificity is required. We concluded that ddPCR is an effective diagnostic technology suitable for both research and clinical use in diagnosing ocular C. trachomatis infections.

## INTRODUCTION

Trachoma, caused by ocular Chlamydia trachomatis infection, remains the leading infectious cause of blindness worldwide. In 1998, a World Health Assembly resolution called for its elimination as a public health problem by 2020. Considerable progress has been made toward achieving this goal by implementing the SAFE strategy: *s*urgery for those with end-stage disease, community-wide mass treatment with *a*ntibiotics, promotion of *f*acial cleanliness, and *e*nvironmental improvement. Since 1998, more than 250 million doses of azithromycin have been donated to trachoma control programs and eight countries in which trachoma was formerly endemic report having met the elimination targets set by the World Health Organization (WHO) (1).

Since the clinical signs of trachoma can persist for months or even years after the elimination of ocular C. trachomatis infection in the community, tests for infection have been valuable in monitoring and optimizing the impact of various control strategies (2–7). Quantitative tests, which measure the bacterial load, have been particularly useful, as they can identify population subgroups in greatest need of treatment (2, 3, 5–8). Nucleic acid amplification tests (NAATs) for the diagnosis and quantitation of clinical C. trachomatis infections are widely used, and a number of quantitative PCR (qPCR) assays have been described (9–12).

Droplet digital PCR (ddPCR) (13, 14) is a next-generation implementation of digital PCR (dPCR) that facilitates the accurate and precise quantitation of nucleic acid targets without the need for calibration curves (15). In the format described here, microfluidic focused-flow droplet generator chips (16) are used to partition a duplex fluorescent-probe-based PCR assay into ∼15,000 highly uniform one-nanoliter-volume reverse (water-in-oil) micelles that are stable at high temperatures. The droplet PCR is performed in a normal thermal cycler. Each droplet in the emulsion is an independent nano-PCR. During the PCR process, the emulsion droplets gel in a manner presumably similar to that reported by Leng et al. (17) to form semisolid microspheres. Post-PCR, droplets are focused into a single file beam of droplets which are flowed through a cytometer under LED excitation that allows highly accurate enumeration of PCR-positive and -negative droplets at high speed.

The digital PCR is formulated in such a way that there are fewer copies of the DNA template than the number of droplets generated, which ensures microfluidic stochastic confinement. The qualitative (positive/negative) endpoint of PCR is determined in each droplet, and given that the distribution of templates to droplets adheres to the Poisson distribution, the counts of positive and negative droplets can be converted into an absolute quantitation of the number of templates in the total PCR volume (15, 18, 19). Almost every PCR-positive droplet in the ddPCR is the endpoint of a reaction that was seeded by a single template molecule, and this, by implication, means that ddPCR is able to reproducibly detect DNA templates at terminal (one-target-per-test) dilution (13, 19, 20). In ddPCR, the absolute concentration of a single target molecule, relative to the PCR reagents, is substantially higher in the nanoliter volume than in conventional microliter-scale PCR. The likelihood of favorable primer-template interactions, and thus, the efficiency, specificity, and sensitivity of ddPCR, is increased in comparison to that of conventional PCR tests (21). Similarly, the fluorescent product is confined to the droplet volume, and so, small changes in fluorescence intensity are more readily detected by photonics equipment than a similar absolute amount of fluorescence would be by conventional qPCR platforms (21). ddPCR is robust against many of the factors that can negatively influence conventional PCR because the DNA template, when confined, is sequestered from cross-reacting DNA templates, inhibitory moieties, and spurious PCR amplicons, such as primer-dimers (20).

The benefits of digital PCR make it a process that has clear utility in the field of quantitative infectious disease diagnostics (22–24). It remains unclear how accurately qPCR methods are able to measure C. trachomatis infectious load in clinical samples in which inhibitory agents, competing DNA, and nonexponential amplification during early PCR cycles can potentially affect changes in PCR amplification efficiency. ddPCR is robust against these factors, which, coupled with its superiority in performance, based on the theoretical arguments presented above, leads to a potentially more accurate and precise assay than preexisting methods. We aimed to evaluate the diagnostic performance of a ddPCR-based C. trachomatis assay and to highlight the utility of the ddPCR method in clinical diagnostics.

## MATERIALS AND METHODS

### ddPCR.

The ddPCR primers targeted the C. trachomatis cryptic plasmid (10) and the Homo sapiens RNase P/MRP 30-kDa subunit (RPP30) gene (25). The sequences of the oligonucleotides used in this study are given in Table 1. ddPCR reaction mixtures were 20-μl volumes that contained final concentrations of 1× ddPCR Supermix (Bio-Rad, Hemel Hempstead, United Kingdom), 0.3 μM each primer and probe (PLASMID/RPP30), and 4.95 μl sample DNA of unknown quantity. Droplet generation and droplet reading for ddPCR were carried out according to the manufacturer's instructions using Bio-Rad reagents. The thermal cycling profile was 95°C for 10 minutes followed by 40 cycles of 95°C for 15 seconds and 60°C for 30 seconds.

**Table 1:** Oligonucleotides used in this study

Target	Primer/probe sequence^*a*^
C. trachomatis plasmid	CAGCTTGTAGTCCTGCTTGAGAGA
	CAAGAGTACATCGGTCAACGAAGA
	FAM-CCCCACCATTTTTCCGGAGCGA-BHQ1
RPP30	AGATTTGGACCTGCGAGCG
	GAGCGGCTGTCTCCACAAGT
	HEX-TTCTGACCTGAAGGCTCTGCGCG-BHQ1

a BHQ, black hole quencher.

### ddPCR data processing.

Raw ddPCR data were collected on the Bio-Rad QX100 instrument using Quantalife software (Bio-Rad) and were then exported for analysis using Perl and R scripts (see data S1 and S2 in the supplemental material). ddPCR data analysis was performed masked to the results of the Amplicor test and the details of clinical phenotypes. The Poisson calculation was used to estimate the number of plasmids/μl of the reaction and confidence intervals (18). For classification purposes, we used the estimated mean concentration of the target and its standard deviation to define the cumulative distribution function (c.d.f.) at *x* = 0. This value describes the probability that the true concentration is less than or equal to zero copies/μl. The classifier ζ was defined as 1 − c.d.f. and describes the probability that the true concentration is greater than zero copies/μl.

We excluded samples from further analysis if the ζ value for the RPP30 assay was below 0.95. Samples for which there was at least 99% droplet occupancy by plasmids were judged as being oversaturated with plasmid templates and potentially contravened assumptions of the Poisson distribution. In these cases, we retested a 1:10 dilution of the specimen and corrected the estimates for this post hoc test.

### Accuracy and precision.

The accuracy (closeness of concentration estimates to the true concentration) and precision (reproducibility of replicate tests) of the ddPCR test were examined by assaying a calibration curve of 17 serial doubling dilutions of a sample of a plasmid vector construct (pCTL12A) that contained the entire sequence of the Chlamydia trachomatis plasmid of an L2 biovar (26). The doubling dilution series was made using a diluent that contained a constant amount of a PCR product containing the RPP30 target sequence. Linear regression was used to determine the *r*^2^, slope, and intercept values for the relationship between ddPCR estimates of plasmid concentration and the standard dilution series.

### Community survey and clinical examination.

A population-based trachoma survey was conducted on four islands of the Bijagós Archipelago of Guinea Bissau in West Africa, where ocular infection with C. trachomatis and trachomatous eye disease are hyperendemic. Trachoma survey methodology has been described previously (27–29). Individuals from randomly selected households in these communities attended for clinical examination and conjunctival sampling between January and March 2012.

A single trained examiner assessed each participant. In this analysis, the WHO simplified grading system was used to assign a trachoma grade to the right and left upper tarsal conjunctivae of each consenting participant.

Ocular swab specimens were collected from a consecutive series of participants. Samples were taken from the left upper tarsal conjunctiva of each participant with Dacron swabs (Fisher Scientific, Loughborough, United Kingdom) using a validated, well-tolerated, standardized protocol (8). Swabs were kept on ice in the field and frozen to −80°C within 8 h of collection. Negative-control specimens were collected in the presence of a participant by passing the swab in front of the participant's eye without making contact.

### DNA extraction.

Swabs were suspended in sterile phosphate-buffered saline (PBS) after being thawed at room temperature. The swabs were then thoroughly vortexed. Liquid was expressed from the swab head against the side of the tube, and swabs were then discarded. DNA was extracted from the PBS suspension using an adapted whole-blood DNA extraction protocol on the QIAxtractor (Qiagen, Manchester, United Kingdom) automated instrument.

### Roche CT/NG Amplicor test.

We used the Roche Amplicor C. trachomatis/Neisseria gonorrhoeae (CT/NG) PCR assay (Roche Molecular Systems, NJ) as the reference test in this evaluation since the clinical specimens came from an epidemiological population sample in which Amplicor had been used to detect C. trachomatis infections. The ddPCR study was performed post hoc. We have used Amplicor widely and extensively in trachoma studies (8, 30–33), and it was the test of choice in a large multicountry study to evaluate trachoma control (34). Others have also evaluated its performance in clinical testing for C. trachomatis infections in numerous studies (35–42). To perform the Amplicor test on purified DNA samples, we diluted 9 μl DNA extract in 94.5 μl of a 1:1 diluent/lysis buffer solution and used 50 μl of this in the standard assay as described previously (28). Positive and negative samples were classified according to the manufacturer's instructions (rev. 3.0; 2010). Amplicor CT/NG testing was performed between April and May 2012. Amplicor “equivocal” samples were confirmed by testing two further aliquots of the same DNA specimen by Amplicor CT/NG according to the manufacturer's instructions (rev. 3.0; 2010). Specimens with at least two of three results with an optical density at 450 nm (OD_450_) of ≥0.2 were classified as positive.

### Diagnostic evaluation.

In the initial analysis, presumptive sample positivity for C. trachomatis was determined when ζ was ≥0.95. A two-by-two comparison against Amplicor CT/NG was performed, and the sensitivity, specificity, positive predictive value (PPV), negative predictive value (NPV), positive likelihood ratio (LR+), negative likelihood ratio (LR−), and respective 95% confidence intervals of the ddPCR test were calculated (43).

We used receiver-operator curve (ROC) analysis to establish the estimated plasmid concentration threshold values at which, compared to Amplicor, (i) sensitivity was maximized, (ii) the optimal balance between specificity and sensitivity was achieved, (iii) specificity was ≥99%, (iv) specificity was ≥99.9%, and (v) specificity was 100%.

Written, informed consent was collected from all study participants. The research committee of the Ministry of Health, Guinea Bissau, and the London School of Hygiene and Tropical Medicine ethics committee granted permission for the study.

## RESULTS

### Visualization of droplet PCR and data processing.

**Fig 1:**
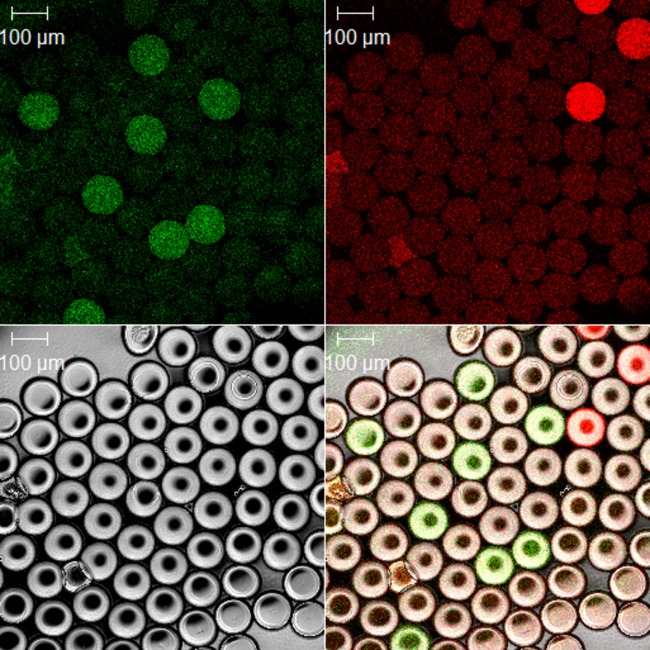
Confocal photomicrograph of ddPCR droplets from a representative C. trachomatis positive sample post-PCR. A bright-field image of droplets is shown at the bottom left. C. trachomatis plasmid PCR-positive droplets are shown on the 6-carboxyfluorescein (FAM) (green) channel (top left), and human RNase P/MRP 30-kDa subunit gene-positive droplets are shown on the HEX (red) channel (top right). A composite of the bright-field, FAM, and HEX channels is shown at the bottom right. All droplets have noticeable baseline fluorescence on both channels. PCR-positive droplets fluoresce with much greater intensity than template-negative droplets. The majority of droplets are PCR negative.

Prior to commencing the clinical evaluation, we visualized under fluorescence microscopy a sample of post-PCR droplets from a human DNA specimen that was infected with C. trachomatis (Fig. 1). We clearly identified numerous droplets that were positive for each of the two targets, thus demonstrating that the assay was functioning correctly. In all successive tests, raw flow cytometric data were processed using automated R scripts to generate a scatterplot of individual droplet fluorescence intensities and a number of technical reports that presented ζ values and estimated concentrations. The open-source R scripts that we developed during this study have been released on a Creative Commons Attribution ShareAlike license, are included in data S1 and S2 in the supplemental material, and can be downloaded for unrestricted free and open use.

### ddPCR accuracy and precision.

**Fig 2:**
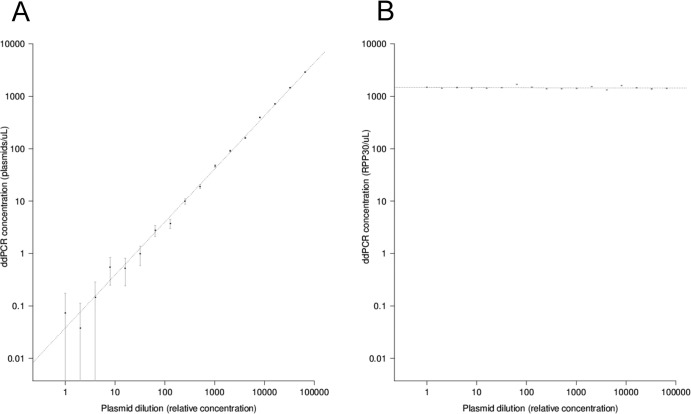
(A) Standard calibration curve of ddPCR concentrations (plasmids/μl) against dilution factors, *r*^2^ = 0.9927, intercept = 0.037 plasmids/μl, slope = 1.01. (B) Standard calibration curve of ddPCR concentrations (human RPP30 copies/μl) against dilution factors, *r*^2^ = 0.02896, intercept = 1,478.1 plasmids/μl, slope = −0.003. All plots are drawn on logarithmic scales.

Using serial dilutions of pCTL12A (C. trachomatis plasmid containing a positive-control sample), we assayed a standard calibration curve, which is shown in Fig. 2A (C. trachomatis plasmid) and B (human RPP30). The *r*^2^ value for the correlation between the dilution factor and the experimentally obtained quantitation was 0.9927. The intercept with the *y* axis was at 0.03776 plasmids/μl, and the slope was 1.01218.

### ddPCR testing of clinical specimens.

**Fig 3:**
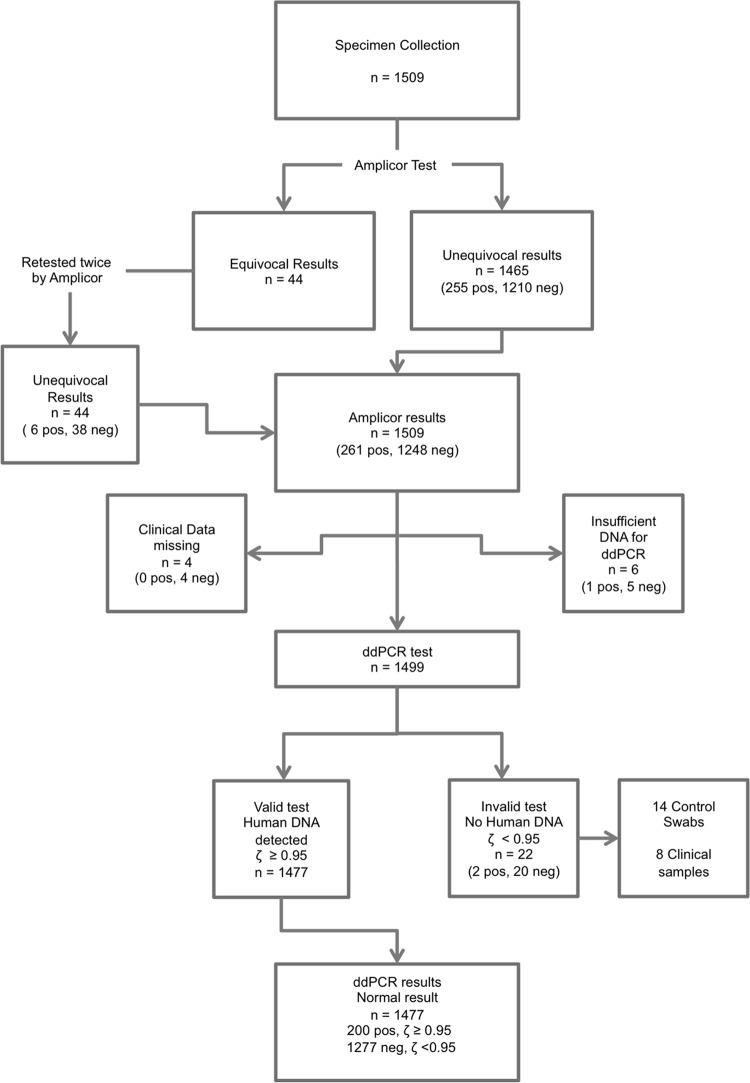
Flow diagram of participant samples in the retrospective validation of ddPCR against the standard Amplicor CT/NG test. Numbers in parentheses refer to Amplicor CT/NG results.

The population sampled was 43% male, with a median age of 13 years (range, 0 to 88 years). We tested 1,495 clinical samples and 14 control swabs. Fourteen hundred seventy-seven specimens were retained for analysis after data entry, cleaning, Amplicor CT/NG, ddPCR, and quality control tests (Fig. 3). The spectrum of clinical signs of trachoma in the population sample is described in Table 2. The association between C. trachomatis quantitation and clinical severity of signs of trachoma is not the focus of this paper and is the subject of a subsequent manuscript (A. R. Last, C. h. Roberts, E. Cassama, M. Nabicassa, D. C. W. Mabey, M. J. Holland, and R. L. Bailey, unpublished data).

**Table 2:** Comparison of Amplicor CT/NG and ddPCR to clinical exam^*a*^

Clinical group^*b*^	No. of subjects in each group	No. (%) with each result by each test			
		Amplicor		ddPCR (ζ ≥ 0.95)	
		Positive	Negative	Positive	Negative
Active trachoma (TF and/or TI)	161	102 (63.3)	59 (36.7)	98 (60.9)	63 (39.1)
TF	158	100 (63.3)	58 (36.7)	96 (60.8)	62 (39.2)
TI	16	12 (75)	4 (25)	12 (75)	4 (25)
TS	343	44 (12.8)	299 (87.2)	26 (7.6)	317 (92.4)
TT	21	6 (28.6)	15 (71.4)	5 (23.8)	16 (76.2)
No trachoma	979	114 (11.6)	865 (88.4)	78 (8)	901 (92)

a Individuals may be included in more than one clinical group.

b TF, trachomatous inflammation, follicular; TI, trachomatous inflammation, intense; TS, trachomatous scarring; TT, trachomatous trichiasis.

At least one C. trachomatis PCR-positive droplet was detected in 305 (20.64%) samples. The median ddPCR droplet count was 14,514 (minimum, 1,171; 1st quartile, 13,720; 3rd quartile, 15,150; maximum, 17,720). The minimum detected quantity of plasmid targets by ddPCR was 0.062 targets/μl (95% confidence interval [CI], 0.209 to 0.184). Droplet occupancy of 99% was reached when the PCR mixture contained ∼5,000 targets/μl (95% CI, 4,868 to 5,217). In specimens that had one or more PCR-positive droplet, the percentage of the total number of droplets that were occupied by a template was uniformly low in both the human and C. trachomatis assays. In the context of the PCR plasmid test, a median of just 0.119% of droplets (minimum, 0.006%; 1st quartile, 0.007%; 3rd quartile, 9.972%; maximum, 98.97%) were occupied by the C. trachomatis plasmid target. Similarly, the percentage of the total number of droplets that was occupied by human RPP30 targets had a median value of 0.015% (minimum, 0.0003%; 1st quartile, 0.007%; 3rd quartile, 0.036%; maximum, 0.757%). The overwhelming majority of all droplets in all tests that were performed contained neither target.

### Comparison to Amplicor CT/NG.

The classification threshold for ddPCR was a ζ value of 0.95. Table 3 shows the sensitivity, specificity, PPV, and NPV of ddPCR compared to those of Amplicor CT/NG at this threshold. Comparisons of Amplicor CT/NG and ddPCR to clinical exam are shown in Table 2. Infection was more likely to be detected in cases of active trachoma than in those without signs of active disease, but there appeared to be no obvious differences between the two tests in the different clinical groups. None of the negative-control swabs taken in the field were positive by Amplicor or by ddPCR for either human or C. trachomatis DNA.

**Table 3:** Diagnostic performance of ddPCR compared to Amplicor CT/NG^*a*^

Result by ddPCR	No. with each result by Amplicor		
	Positive	Negative	Total
Positive	189	11	200
Negative	69	1,208	1,277
Total	258	1,219	1,477

a ddPCR-positive samples defined by a ζ of ≥0.95. Sensitivity was 73.3% (95% CI, 67.9 to 78.7%). Specificity was 99.1% (95% CI, 98.6 to 99.6%). PPV was 94.5% (95% CI, 91.3 to 97.7%). NPV was 94.6% (95% CI, 93.4 to 95.8%). LR+ was 81.45 (95% CI, 45 to 147). LR− was 0.27 (95% CI, 0.22 to 0.33).

ROC-determined sensitivity, specificity, NPV, PPV, LR+, and LR− at different thresholds are shown in Table 4. The overall diagnostic efficiency of the ddPCR assay compared to that of Amplicor CT/NG, defined by the area under the curve (AUC), was 90.8%. The AUC gives the probability that a randomly selected positive specimen will be ranked higher than a randomly selected negative specimen and, as such, is a general indicator of the performance, or “predictiveness,” of the assay (44).

**Table 4:** ROC-determined sensitivity, specificity, PPV, NPV, LR+, and LR− at different ddPCR concentration threshold values

Threshold value (plasmids/μl)	Sensitivity (%)	Specificity (%)	PPV (%)	NPV (%)	LR+	LR−
0.0707	83.7	93.7	73.7	96.5	13.25	0.17
0.0732	82.6	95.0	77.7	96.3	16.50	0.18
0.206	74.4	99.0	94.11	94.8	75.60	0.26
0.944	61.2	99.9	99.4	92.4	746.52	0.39
4.122	52.7	100	100	90.1	Infinity	0.47

**Fig 4:**
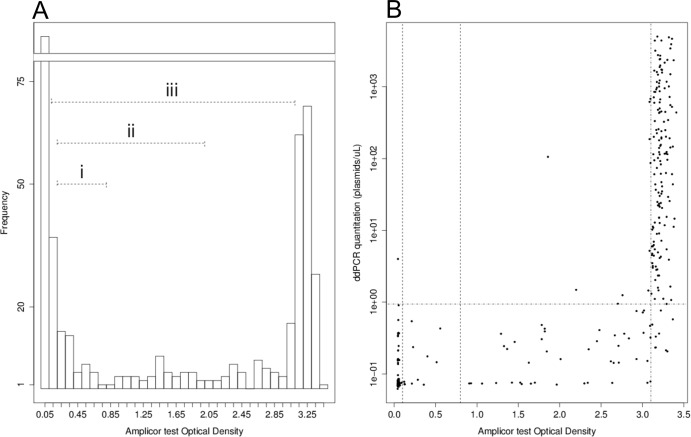
(A) Distribution of Amplicor CT/NG test OD_450_ values, showing (i) the most-recent Amplicor CT equivocal zone, (ii) the earlier Amplicor equivocal zone, and (iii) the broad equivocal zone identified using the observations of this study. (B) Amplicor CT test OD_450_ (*x* axis) against C. trachomatis plasmid quantity (*y* axis), as determined by ddPCR. OD_450_ values of 0.1, 0.8, and 3.1 are indicated. ddPCR values of zero are assigned an arbitrarily low (0.001 plasmids/μl) value. Positive ddPCR with high estimates of the infectious load are infrequent when the Amplicor CT/NG OD_450_ is <3.1.

Figure 4A shows the distribution of Amplicor CT/NG OD_450_ values in the sample population data. There are clear negative and positive populations at the extremes of the OD_450_ range, and we used the data presented in Fig. 4 to define an extended area of intermediate values (0.8 < OD_450_ < 3.1) which is more extensive than those described in the Amplicor literature and for which the classification is less clear. Figure 4B shows correlations between the Amplicor CT/NG OD_450_ values and the ddPCR estimates of plasmid concentrations. Specimens that had intermediate OD_450_ values (0.8 < OD_450_ < 3.1) were unlikely to have high plasmid loads according to ddPCR, and the median concentration of C. trachomatis plasmid in an Amplicor CT/NG-positive, ddPCR-positive sample was just 0.24 plasmids/μl (minimum, 0.071; 1st quartile, 0.076; 3rd quartile, 0.458; maximum, 682.9) when the Amplicor CT/NG optical density signal at 450 nm was between 0.8 and 3.1. Conversely, samples were much more likely to have high plasmid loads when the Amplicor CT/NG OD_450_ value was in the clear positive range (OD_450_ ≥ 3.1). The median plasmid concentration of specimens in this group was 94.94 plasmids/μl (minimum, 0.206; 1st quartile, 7.188; 3rd quartile, 608.900; maximum, 5,029.000).

## DISCUSSION

Recent studies in several countries have shown that a high prevalence of follicular trachoma (TF) can persist for months or years in communities in which the prevalence of ocular C. trachomatis has been reduced to a very low level, or even eliminated, following mass treatment (32, 45, 46). Current recommendations are to continue mass treatment with azithromycin until the prevalence of TF in children ages 1 to 9 years is below 5%, but this can lead to repeated rounds of mass treatment for communities in which few, if any, individuals are infected. Basing the decision to stop mass treatment on the prevalence of C. trachomatis infection would save resources and reduce the risk of selecting for macrolide-resistant strains of important pathogens, such as Streptococcus pneumoniae (47). As the prevalence of infection falls, so does the positive predictive value of a test for infection. It is therefore particularly important, in designing a test that will be used to determine that infection has been eliminated from a community, to ensure that it is highly specific. Based on the evidence of a comparison to a well-validated reference test, Amplicor CT/NG, the ddPCR assay fulfills this criterion.

The standard calibration curve is a simultaneous test in which a single metric, the coefficient of determination (the *r*^2^ value), is used to describe the accuracy and intraoperator precision of PCR assays. Using a serial dilution calibration series, we found that the ddPCR test is a highly precise and accurate (*r*^2^ = ∼0.9927) assay that can detect and quantify chlamydial plasmids across a wide range of concentrations. Sampling error in the ddPCR test was assessed by examination of the 95% confidence intervals (Fig. 2) of the concentration estimates. The increase in sampling error that we observed at lower dilutions clearly reflected the stochastic sampling process that takes place when an aliquot is taken from a larger specimen volume. Slight variation in the levels of RPP30 that were detected (Fig. 2B) was able to be explained by error introduced by liquid-handling procedures.

We chose to use a confidence measure (ζ) to classify the results of ddPCR tests. This value is derived from the normal probability distribution of the ddPCR concentration and describes the probability that the true concentration is above zero copies/μl. For the classification of specimens as positive for the PCR targets of either the human or C. trachomatis assays, we initially chose an arbitrary cutoff value for ζ of 0.95. At this threshold, there is 95% confidence that the true target concentration is not zero. In using a threshold of ζ of 0.95, we define a highly specific assay in which there is a theoretical maximum probability of false-positive results in any given test that is less than 5%.

We took a standard approach to diagnostic evaluation and used ROC analysis to compare the ddPCR test results to a set of Amplicor CT/NG data that had been classified according to the most recent version of the manufacturer's protocol (rev. 3.0; 2010). In this case, we used the estimated concentration of plasmids per microliter of the PCR mixture as the classifier. This value, which is proportional to ζ, is the biologically meaningful quantitative output of the ddPCR assay. We found that ddPCR performed extremely well in this population (ROC AUC was above 90%; Amplicor CT/NG estimated prevalence was ∼17.47%). Table 4 shows how differing threshold values of the ddPCR classifier can be used to optimize the assay (highly sensitive/highly specific/balanced) for use in different prevalence settings.

The evidence from our direct comparison to Amplicor CT/NG indicated that the ddPCR test was less sensitive (maximally 83.7%) than Amplicor CT/NG. We acknowledge that our ddPCR C. trachomatis test may be inferior to Amplicor CT/NG both in general terms and particularly with respect to sensitivity. The observation of lower-than-expected sensitivity is similar to the findings of a previous study (22), in which a human cytomegalovirus (CMV) ddPCR assay was found to be less sensitive than a qPCR assay for the same target. These findings are surprising given the theoretical technological advantage of ddPCR over conventional microliter-scale fluidic PCR. If false-positive classification by the Amplicor CT/NG assay had occurred, the result would be a false indication of low sensitivity in ddPCR. Previous studies have used discrepant analysis to resolve divergent results between the index and reference tests (36, 37, 41, 42), and conditionally independent tests to arbitrate discrepancies (with proven high sensitivity), such as the Gen-Probe Aptima test, (48) are available. This test is based on C. trachomatis RNA detection, and we did not have access to RNA specimens or any other biological material from the individuals who participated in the study. The use of a third DNA-based test, such as qPCR, would not be conditionally independent, and the validity of arbitration by discrepant analysis remains controversial in any case (49, 50).

The diagnostic classifiers in the qualitative Amplicor CT/NG (OD_450_) and quantitative ddPCR (estimated target concentration) tests are both continuous variables. Although the Amplicor CT/NG OD_450_ value is not thought to be an accurate estimate of the DNA targets contained in the original specimen, this value is the continuous quantitative measure that is used to define boundaries for sample positivity. Changing the critical threshold value at which positive/negative classification is made can affect the outcome of comparative diagnostic evaluations, as demonstrated in Table 4. The choice of threshold in the reference is therefore a critical determinant of the diagnostic evaluation because misclassification by the reference has deleterious downstream effects on the estimation of the diagnostic performance of the comparative test. Figure 4A shows how a large number (*n* = 172) of data points fell within what we describe as an expanded equivocal zone in the Amplicor CT/NG data (0.1 to 3.1), wherein the classification would change if different Amplicor CT/NG thresholds were chosen.

In the product insert for the Amplicor CT/NG test (rev. 3.0; 2010), the manufacturer recommended a cutoff threshold for Amplicor CT of 0.8 OD_450_ units, with triplicate testing indicated for all equivocal values between 0.2 and 0.8 in the initial test. Earlier versions (available online at http://www.fda.gov/downloads/BiologicsBloodVaccines/SafetyAvailability/TissueSafety/ucm100246.pdf) present figures of data and recommend a much wider equivocal zone for which testing by alternate methods would be required (0.2 to 2.0). We believe that the most recent guidelines limit the equivocal range to the area in which false positives are most likely (OD_450_ = 0.2 to 0.8) instead of the area in which they are possible (OD_450_ = 0.2 to ∼3) and that this may increase the cost-effectiveness of C. trachomatis screening, as these guidelines require fewer replicate or alternate tests than the earlier guidelines. The result of these measures is the implementation of a highly sensitive test, but with the consequence of a significant number of false-positive results. The Amplicor test guidelines were optimized for use in testing for genitourinary tract infections, for which the risk of false-negative results would be minimized. While Amplicor CT/NG has been validated and widely used in testing ocular samples (8, 30–34), any misclassification that has occurred in this study might be the result of using Amplicor classification cutoff values that are not appropriate to the ocular specimens. Nonetheless, when we chose a ddPCR threshold (∼0.07 plasmids/μl) that maximized sensitivity, 42 Amplicor-positive but ddPCR-negative specimens remained. We anticipate that some proportion of these is attributable to misclassification by Amplicor CT/NG testing.

A second source of error in the sensitivity estimate is shown in Fig. 4B. We assessed samples that were ddPCR and Amplicor CT/NG (i.e., OD_450_ of >0.8) positive and found that the ddPCR estimate of the plasmid concentration was uniformly low (median, 0.24 plasmids/μl) when the Amplicor CT/NG OD_450_ value was in the range of 0.2 to 3.1, which we defined as the “expanded equivocal zone.” Samples in the Amplicor CT-positive range (OD_450_ > 3.1) had much higher concentration estimates by ddPCR (median, 87.99 plasmids/μl). We take these observations to indicate that a significant number of the discrepancies are the result of sampling error (which affects the performance of most NAATs) and/or suboptimal performance when presented with a specimen that has a low target sequence load.

We developed a highly specific test that is widely applicable for C. trachomatis testing and may be useful in monitoring infection post-mass drug administration (MDA). We aim to maintain high specificity, which we can define by a stringent threshold value of ζ of ≥0.95. Reducing the threshold value of ζ would lead to dramatic increases in sensitivity, but the confidence with which we would accept a sample as positive is reduced, and this might lead to overdiagnosis. This is particularly important when or if MDA treatment decisions are informed by a test for current ocular chlamydial infection rather than by clinical signs of trachoma alone (51).

Previous studies by us and others have suggested that there is a threshold of C. trachomatis infection prevalence and intensity below which the infection will spontaneously disappear from a population—the so-called Allee effect (5, 45, 46). A test that accurately estimates the bacterial load, such as the ddPCR assay, may be especially valuable in determining when this threshold has been reached and it is therefore safe to discontinue MDA.

The ddPCR technology has great utility as a diagnostic. Unlike most other NAATs, this method requires no external or internal calibration yet delivers a highly accurate estimation of target load. The inclusion of a human target allows a system of internal control that indicates (but does not discriminate between) experimental failures that are due to either technical PCR failure or the absence of a sample of human origin in the specimen.

We have validated a highly specific quantitative ddPCR assay and applied it to population monitoring of ocular C. trachomatis infections. This method can rapidly be adapted for use in the detection of sexually transmitted C. trachomatis infections, for which a higher sensitivity than that which we have estimated would be desirable. This study represents an early example of the usefulness of this next-generation digital PCR method in diagnosing infections in clinical specimens within the arena of infectious diseases.

## Supplementary Material

Supplemental material
