# Supplementary material for: Development and Evaluation of a Next-Generation Digital PCR Diagnostic Assay for Ocular Chlamydia trachomatis Infections
Source: J Clin Microbiol. 2013 Jul;51(7):2195–203. doi: 10.1128/JCM.00622-13 (PMC3697714; doi:10.1128/JCM.00622-13)
Supplement: Supplemental material [file supp_51_7_2195__index.html]

Development and Evaluation of a Next-Generation Digital PCR Diagnostic Assay for Ocular Chlamydia trachomatis Infections — Supplemental material 

# Development and Evaluation of a Next-Generation Digital PCR Diagnostic Assay for Ocular Chlamydia trachomatis Infections

## 

**Files in this Data Supplement:**

- Supplemental file 1 -

  Data S1 (Perl script for processing of droplet digital PCR data) and S2 (R script for processing of droplet digital PCR data)

  PDF, 129K
