# Supplementary material for: Development and Evaluation of a Next-Generation Digital PCR Diagnostic Assay for Ocular Chlamydia trachomatis Infections
Source: J Clin Microbiol. 2013 Jul;51(7):2195–203. doi: 10.1128/JCM.00622-13 (PMC3697714; doi:10.1128/JCM.00622-13)
Supplement: Supplemental material [file JCM.00622-13_zjm999092624so1.pdf]

TITLE

Clinical evaluation of the diagnostic accuracy of a droplet digital PCR assay for the detection of Chlamydia trachomatis infections.

SUPPLEMENTARY DATA : Perl and R scripts for data analysis and interpretation

Chrissy h. Roberts[1#], Anna Last[1], Sandra Molina-Gonzalez[1], Eunice Cassama[4], Robert Butcher[1], Meno Nabicassa[4], Elizabeth McCarthy[2], Sarah E Burr[1,3], David C. Mabey[1], Robin L. Bailey[1] and Martin J. Holland[1].

1 Clinical Research Department, London School of Hygiene and Tropical Medicine, London, UK

2 Department of Immunology, London School of Hygiene and Tropical Medicine, London, UK

3 Medical Research Council Unit, The Gambia

4 Programa Nacional de Saude de Visao, Ministerio de Saude Publica, Bissau, Guinea Bissau

# Corresponding author.
Mailing address :
Clinical Research Department, London School of Hygiene and Tropical Medicine, Keppel St. London, UK WC1E 7HT
Phone : +44 (0) 20 7927 2913
E-mail : chrissy.roberts@lshtm.ac.uk

CONTENTS

S1 : Perl script “drops.pl”
S2 : R script “dropletR_man_thresholds.R”

INTRODUCTION

Supplements S1 and S2 are software scripts that we developed as tools to assist in the processing of raw data from the Quantalife software. Scripts are small computer programmes that act like a series of instructions to a software environment and order it to execute a series of tasks. These scripts can be used or modified (with correct attribution of the source) by anyone and for any purpose as they are released under the Creative Commons Attribution-ShareAlike 3.0 Unported License.

Raw data is read from ~/in and results are written to ~/out, where ~/ is the current working directory.
Please ensure that you make the in and out sub-directories before running the script for the first time

To run these scripts you will need a copy of R and Perl.
Perl is usually installed as standard on unix machines (including Apple Mac, Ubuntu and so on). For Windows PCs, we recommend that you use "strawberry perl" which is available as a free download from http://strawberryperl.com/
You may also need to install an X windows system (such as XQuartz on a Mac, Xming server for Windows)

Before using these scripts, you should copy the script text from this file to two new files, as indicated below.

HOW TO USE THE SCRIPTS

To run the scripts, open a terminal (command prompt) in the directory that contains the two script files and the ~/in and ~/out directories. Then at the prompt ($ or C:/) type

```
perl drops.pl [enter]
```

Then follow the onscreen commands.

S1 : drops.pl : Data processing for droplet digital PCR data (ddPCR v1.01)

This Perl script is designed to process data from the Bio-Rad DX100 platform into a format that is more compatible with R than are the raw files that are output from the quantalife software.

The quantalife software is able to export raw fluorescence data. After performing a run, you should go to the setup screen and select options, then export raw data. This will generate one CSV file for each well of the sample plate. Each file has two columns, the first is the fluorescence intensity for the FAM channel, the second the same for VIC/HEX. Each row is a separate droplet. This script will collect individual files from a subfolder "in" and will process them in to a single output file "ddPCRout.txt" in the current working directory which contains a concatenation of the total data, with well names appended to the FI data. You will be asked for a plate number and this will be appended to downstream workflows. This script will automatically spawn an R session and run the accompanying R script.

Lines starting with a hash (#) contain notes, explanations and annotations to the script.

The drops.pl script starts on the next page, copy the indicated text (which is in courier font) to a text file and save it with the name drops.pl

```
#!/usr/bin/env perl
# The preceding line must be the first line of the drops.pl file

use warnings;
use Cwd;
#get current working directory
my $dir = getcwd();
print
"##########################################################################################
#   TITLE : drops.pl : Data processing for droplet digital PCR data (ddPCR v1.01)
##########################################################################################
#                                   Open source license
##########################################################################################
# To attribute this work, you must cite the name of the original source, the name of
# the author (Chrissy h. Roberts) and their contact details
# (chrissyhroberts\@yahoo.co.uk). This work is licensed under the Creative Commons
# Attribution-ShareAlike 3.0 Unported License.
# To view a copy of this license, visit http://creativecommons.org/licenses/by-sa/3.0/
# or send a letter to Creative Commons, 444 Castro Street, Suite 900, Mountain View,
# California, 94041, USA.
##########################################################################################\n\n";
print "\n$dir\n";
#get plate number/name
print"\n";
print "what is the plate number/name : ";
chomp(my $platenumber = <STDIN>);
print "\n\nthanks, the plate number is $platenumber\n\n";
#get method for calling positive/negative droplets
$test = 1;
#get list of files in the cwd/in folder (these should be CSV files that were exported from
Quantalife software)
```

```
@files = <$dir/in/*>;
print"files: ".@files."\n";
#create new file for output data
open OUT,">","ddPCRout.txt";
my $colour = 1;
#for each file, add current plate and well names to fluorescence intensity data and write output
to file
select OUT;
print "SAMPLE\tFAM\tVIC\n";
foreach (@files) {
my $currentfile = $_;
open IN,   "<","$_";
my @lines = <IN>;
foreach(@lines ){
chomp;
if ($_ =~ /Amplitude/){next};
s/,/\t/g;
$currentfile = substr($currentfile,-17,17);
$currentfile = substr($currentfile,0,3);
print "$platenumber$currentfile\t$_";
}
close IN;
}
select STDOUT;
# spawn R session and run script
print "\n\n\nProcessed raw files, performing R script...\n\n\n";
if($test==1){system "R CMD BATCH dropletR_man_thresholds.R"};
print"Job DONE.\n\n\n";
##########################################################################################
# End of SCRIPT drops.pl       This should be the last line of the file
##########################################################################################
```

S2 : dropletR_man_thresholds.R : Data processing for droplet digital PCR data (ddPCR v1.01)

This R script takes the output (ddPCRout.txt) of the drops.pl script as the input, then performs the following

1: The user chooses the threshold values for each sample. This is done via a simple point and click method. A scatterplot will be drawn for each specimen. Use your pointing device to choose the intersection of the x (FAM) and y (HEX/VIC) thresholds.

2: R uses the raw fluorescence intensity data in ddPCRout.txt and the threshold values for each specimen to estimate the quantities of the targets at each of the two fluorophores (FAM and HEX/VIC)

The data output contains a number of items

000_average_beadcounts.pdf	Plot of droplet counts for the samples
000_average_fam_FI.pdf	Plot of average Fam FI for positive and negative populations of each sample
000_average_vic_FI.pdf	Plot of average VIC/HEX FI for positive and negative populations of each sample
000_concentration_fam.pdf	Summary of FAM channel concentration per swab (copies/uL)
000_concentration_vic.pdf	Summary of VIC/HEX channel concentration per swab (copies/uL)
000_results_full.txt	Table containing all calculated data
000_results_concise.txt	Table containing concise data
000_A01.pdf	Scatterplot for FI values, well A01
000_A02.pdf	Scatterplot for FI values, well A02
...	...
platesummary.pdf	Scatterplot of all data points (can be a big file)

The most important file is probably the 000_results_concise.txt file. This contains a simple summary of the really important data points

This concise report has the following fields

Var1	: The name of the specimen (plate number and well location)
endogenouscontrolresult	: Was the endogenous control positive (VIC zeta score at least 0.95)?
targetresult	: Was the sample positive for the target (FAM zeta at least 0.95)?
concentration_fam_average_copies_uL	: Mean average estimated concentration target (copies/uL of the PCR mix)
concentration_fam_low_copies_uL	: Lower 95% CI estimated concentration target (copies/uL of the PCR mix)
concentration_fam_high_copies_uL	: Upper 95% CI estimated concentration target (copies/uL of the PCR mix)
concentration_vic_average_copies_uL	: Mean average estimated concentration endogenous control (copies/uL of the PCR mix)
concentration_vic_low_copies_uL	: Lower 95% CI estimated concentration endogenous control (copies/uL of the PCR mix)
concentration_vic_high_copies_uL	: Upper 95% CI estimated concentration endogenous control (copies/uL of the PCR mix)
areaabovezerofam	: Zeta value (FAM)
areaabovezerovic	: Zeta value (VIC)
Freq	: Total number of assayed droplets
volume	: Total volume of PCR mixture assayed
famposvicneg	: Count of FAM positive, VIC negative droplets
famnegvicpos	: Count of FAM negative, VIC positive droplets
famposvicpos	: Count of FAM positive, VIC positive droplets
famnegvicneg	: Count of FAM negative, VIC negative droplets
famthreshold	: Threshold value of FAM fluorescence intensity for positive droplet classification
victhreshold	: Threshold value of VIC fluorescence intensity for positive droplet classification

The full report contains more detailed information and shows how copies/uL eastimates relate to the original specimen.
In most cases, it will not be necessary to refer to the full report.

The dropletR_man_thresholds.R script starts on the next page, copy the indicated text (in courier font) to a text file and save it with the name dropletR_man_thresholds.R

```
#This should be the first line of the script


###########################################################################################
#                                  Open source license                                    #
###########################################################################################
# To attribute this work, you must cite the name of the original source, the name of     #
# the author (Chrissy h. Roberts) and their contact details                               #
# (chrissyhroberts@yahoo.co.uk). This work is licensed under the Creative Commons        #
# Attribution-ShareAlike 3.0 Unported License.                                            #
# To view a copy of this license, visit http://creativecommons.org/licenses/by-sa/3.0/   #
# or send a letter to Creative Commons, 444 Castro Street, Suite 900, Mountain View,     #
# California, 94041, USA.                                                                 #
###########################################################################################

delete.all <- function()
rm(list=ls(pos=.GlobalEnv), pos=.GlobalEnv)
delete.all()
ls()
cwd <-getwd()
cwd
cat(cwd,"/out/",sep="")
outwd <-paste(cwd,"/out/",sep="")
path <- cwd
outpath <- outwd

#READ IN PLATE
PLATE=read.table(file.path(path,"ddPCRout.txt"),h=T)

#PLOT TOTAL DATA FAM VS VIC
pdf(file.path(outpath,"platesummary.pdf"))
```

```
plot(PLATE$FAM,PLATE$VIC,xlab="PLASMID",ylab="HURNASE",
xlim=c(0,max(PLATE$FAM)+100),ylim=c(0,max(PLATE$VIC)+100),pch = 46)
dev.off()

#define some empty values
samples<-table(PLATE$SAMPLE)
samples<-as.data.frame(samples)
samples$chlamydia_test<-NA
samples$FAILS<-NA
samples$concentration_fam_per_swab<-NA
samples$concentration_fam_per_swab_low<-NA
samples$concentration_fam_per_swab_high<-NA
samples$concentration_vic_per_swab<-NA
samples$concentration_vic_per_swab_low<-NA
samples$concentration_vic_per_swab_high<-NA


# Get sample by sample thresholds and gates

rownumbers <- c(1:length(samples$Var1))
rownumbers <- c(1:length(samples$Var1))

for (i in rownumbers) {

#get well name
coordinate <- samples$Var1[i]

#subset based on well name
currentsubset <- subset(PLATE,PLATE$SAMPLE==coordinate)
x11()
```

```
plot(PLATE$FAM[PLATE$SAMPLE==samples$Var1[i]],PLATE$VIC[PLATE$SAMPLE==samples$Var1[i]],xlab="PLA
SMID",ylab="HURNASE", xlim=c(0,max(PLATE$FAM)+100),ylim=c(0,max(PLATE$VIC)+100),pch =
46,main=samples$Var1[i])
thresholds<-locator(n=1)
dev.off()

samples$famthreshold[i]<-thresholds$x[1]
samples$victhreshold[i]<-thresholds$y[1]

#count fam pos only
samples$famposvicneg[i]<-((length(which(currentsubset$FAM > samples$famthreshold[i] &
currentsubset$VIC < samples$victhreshold[i]) )))
if (is.na(samples$famposvicneg[i])){samples$famposvicneg[i]<-0}


#count vic pos only
samples$famnegvicpos[i]<-((length(which(currentsubset$FAM < samples$famthreshold[i] &
currentsubset$VIC > samples$victhreshold[i]) )))
if (is.na(samples$famnegvicpos[i])){samples$famnegvicpos[i]<-0}

#count double pos only
samples$famposvicpos[i]<-((length(which(currentsubset$FAM > samples$famthreshold[i] &
currentsubset$VIC > samples$victhreshold[i]) )))
if (is.na(samples$famposvicpos[i])){samples$famposvicpos[i]<-0}

#count double neg only
samples$famnegvicneg[i]<-((length(which(currentsubset$FAM < samples$famthreshold[i] &
currentsubset$VIC < samples$victhreshold[i]) )))
if (is.na(samples$famnegvicneg[i])){samples$famnegvicneg[i]<-0}

pdf(paste(outpath,samples$Var1[i], ".pdf", sep=""))
```

```
print (samples$Var1[i])
plot(PLATE$FAM[PLATE$SAMPLE==samples$Var1[i]],PLATE$VIC[PLATE$SAMPLE==samples$Var1[i]],xlab="PLA
SMID",ylab="HURNASE", xlim=c(0,max(PLATE$FAM)+100),ylim=c(0,max(PLATE$VIC)+100),pch =
46,main=samples$Var1[i])
abline(v=samples$famthreshold[i], col="PINK", lty=3)
abline(h=samples$victhreshold[i], col="PINK", lty=3)
dev.off()


#CALCULATE AVERAGE POS FI AND AVERAGE NEG FI


currentsubset_2<-subset(currentsubset,(currentsubset$FAM > samples$famthreshold[i]))
samples$ave_fi_fam_positives[i]<-mean(currentsubset_2$FAM)


currentsubset_2<-subset(currentsubset,(currentsubset$FAM < samples$famthreshold[i]))
samples$ave_fi_fam_negatives[i]<-mean(currentsubset_2$FAM)


currentsubset_2<-subset(currentsubset,(currentsubset$VIC > samples$victhreshold[i]))
samples$ave_fi_vic_positives[i]<-mean(currentsubset_2$VIC)



currentsubset_2<-subset(currentsubset,(currentsubset$VIC < samples$victhreshold[i]))
samples$ave_fi_vic_negatives[i]<-mean(currentsubset_2$VIC)
```

```
#remove currentsubset for tidiness

rm(currentsubset,currentsubset_2)
}

# quality assessment of FI values from positives and negatives

platewide_ave_fam_pos_FI<-mean(samples$ave_fi_fam_positives,na.rm=T)
platewide_ave_fam_neg_FI<-mean(samples$ave_fi_fam_negatives,na.rm=T)
platewide_ave_vic_pos_FI<-mean(samples$ave_fi_vic_positives,na.rm=T)
platewide_ave_vic_neg_FI<-mean(samples$ave_fi_vic_negatives,na.rm=T)
sdplatewide_ave_fam_pos_FI<-sd(samples$ave_fi_fam_positives,na.rm=T)
sdplatewide_ave_fam_neg_FI<-sd(samples$ave_fi_fam_negatives,na.rm=T)
sdplatewide_ave_vic_pos_FI<-sd(samples$ave_fi_vic_positives,na.rm=T)
sdplatewide_ave_vic_neg_FI<-sd(samples$ave_fi_vic_negatives,na.rm=T)
if (is.na(sdplatewide_ave_fam_neg_FI)){sdplatewide_ave_fam_neg_FI<-platewide_ave_fam_neg_FI}
if (is.na(sdplatewide_ave_fam_pos_FI)){sdplatewide_ave_fam_pos_FI<-platewide_ave_fam_pos_FI}
if (is.na(sdplatewide_ave_vic_neg_FI)){sdplatewide_ave_vic_neg_FI<-platewide_ave_vic_neg_FI}
if (is.na(sdplatewide_ave_vic_pos_FI)){sdplatewide_ave_vic_pos_FI<-platewide_ave_vic_pos_FI}

#plot vic average FI for positives and negatives with platewide 99%CI
pdf(file.path(outpath,"000_average_vic_FI.pdf"))

plot(samples$ave_fi_vic_positives,pch=46,col="red",cex=5,ylim=c(0,(platewide_ave_vic_pos_FI+(6*s
dplatewide_ave_vic_pos_FI))),ylab="000_average vic FI, pos/neg and 99.9% CI",xaxt="n")
points(samples$ave_fi_vic_negatives,pch=46,cex=5)
abline(h=(platewide_ave_vic_pos_FI+2.59*sdplatewide_ave_vic_pos_FI), col="PINK", lty=1)
abline(h=(platewide_ave_vic_pos_FI-2.59*sdplatewide_ave_vic_pos_FI), col="PINK", lty=1)
abline(h=(platewide_ave_vic_neg_FI+2.59*sdplatewide_ave_vic_neg_FI), col="PINK", lty=1)
```

```
abline(h=(platewide_ave_vic_neg_FI-2.59*sdplatewide_ave_vic_neg_FI), col="PINK", lty=1)
axis(1,rownumbers,labels=samples$Var1,cex.axis=0.4,las=2)
grid(nx=100,ny=NULL)
dev.off()

#plot fam average FI for positives and negatives with platewide 99%CI

pdf(file.path(outpath,"000_average_fam_FI.pdf"))

plot(samples$ave_fi_fam_positives,pch=46,col="red",cex=5,ylim=c(0,(platewide_ave_fam_pos_FI+1000
)),ylab="average fam FI, pos/neg and 99% CI",xaxt="n")
points(samples$ave_fi_fam_negatives,pch=46,cex=5)
abline(h=(platewide_ave_fam_neg_FI+2.59*sdplatewide_ave_fam_neg_FI), col="PINK", lty=1)
abline(h=(platewide_ave_fam_neg_FI-2.59*sdplatewide_ave_fam_neg_FI), col="PINK", lty=1)
abline(h=(platewide_ave_fam_pos_FI+2.59*sdplatewide_ave_fam_pos_FI), col="PINK", lty=1)
abline(h=(platewide_ave_fam_pos_FI-2.59*sdplatewide_ave_fam_pos_FI), col="PINK", lty=1)
axis(1,rownumbers,labels=samples$Var1,cex.axis=0.4,las=2)
grid(nx=100,ny=NULL)
dev.off()


#assign warnings based on FI average values and 95% CI platewide
samples$vicwarningspositives<-NA
for (i in rownumbers) {if (is.na(samples$ave_fi_vic_positives[i])){next}else{if
(samples$ave_fi_vic_positives[i]>platewide_ave_vic_pos_FI+(2.59*sdplatewide_ave_vic_pos_FI)){sam
ples$vicwarningspositives[i]<-"vic average FI positive value is greater than 99% upper CI limit
for the plate"}}}
for (i in rownumbers) {if
(is.na(samples$ave_fi_vic_positives[i])){next}else{if(samples$ave_fi_vic_positives[i]<platewide_
ave_vic_pos_FI-(2.59*sdplatewide_ave_vic_pos_FI)){samples$vicwarningspositives[i]<-"vic average
FI positive value is lesser than 99% upper CI limit for the plate"}}}
```

```
for (i in rownumbers) {if
(is.na(samples$ave_fi_vic_positives[i])){samples$vicwarningspositives[i]<-"ENDOGENOUS CONTROL
FAILED"}}



samples$vicwarningsnegatives<-NA
for (i in rownumbers) {if (is.na(samples$ave_fi_vic_negatives[i])){next}else{if
(samples$ave_fi_vic_negatives[i]>platewide_ave_vic_neg_FI+(2.59*sdplatewide_ave_vic_neg_FI)){sam
ples$vicwarningsnegatives[i]<-"vic average FI negative value is greater than 99% upper CI limit
for the plate"}}}
for (i in rownumbers) {if (is.na(samples$ave_fi_vic_negatives[i])){next}else{if
(samples$ave_fi_vic_negatives[i]<platewide_ave_vic_neg_FI-
(2.59*sdplatewide_ave_vic_neg_FI)){samples$vicwarningsnegatives[i]<-"vic average FI negative
value is lesser than 99% upper CI limit for the plate"}}}
for (i in rownumbers) {if
(is.na(samples$ave_fi_vic_positives[i])){samples$vicwarningsnegatives[i]<-"NO BASELINE : DROPLET
SATURATION, SYSTEM ERROR OR NO SAMPLE"}}


samples$famwarningspositives<-NA
for (i in rownumbers) {if
(is.na(samples$ave_fi_fam_positives[i])){next}else{if(samples$ave_fi_fam_positives[i]>platewide_
ave_fam_pos_FI+(2.59*sdplatewide_ave_fam_pos_FI)){samples$famwarningspositives[i]<-"fam average
FI positive value is greater than 99% upper CI limit for the plate"}}}
for (i in rownumbers) {if
(is.na(samples$ave_fi_fam_positives[i])){next}else{if(samples$ave_fi_fam_positives[i]<platewide_
ave_fam_pos_FI-(2.59*sdplatewide_ave_fam_pos_FI)){samples$famwarningspositives[i]<-"fam average
FI positive value is lesser than 99% upper CI limit for the plate"}}}

samples$famwarningsnegatives<-NA
```

```
for (i in rownumbers) {if (is.na(samples$ave_fi_fam_negatives[i])){next}else{if
(samples$ave_fi_fam_negatives[i]>platewide_ave_fam_neg_FI+(2.59*sdplatewide_ave_fam_neg_FI)){sam
ples$famwarningsnegatives[i]<-"fam average FI negative value is greater than 99% upper CI limit
for the plate"}}}
for (i in rownumbers) {if (is.na(samples$ave_fi_fam_negatives[i])){next}else{if
(samples$ave_fi_fam_negatives[i]<platewide_ave_fam_neg_FI-
(2.59*sdplatewide_ave_fam_neg_FI)){samples$famwarningsnegatives[i]<-"fam average FI negative
value is lesser than 99% upper CI limit for the plate"}}}
for (i in rownumbers) {if
(is.na(samples$ave_fi_fam_negatives[i])){samples$famwarningsnegatives[i]<-"NO BASELINE : DROPLET
SATURATION, SYSTEM ERROR OR NO SAMPLE"}}


#PLOT BEAD COUNTS FOR ALL SAMPLES
platewide_average_beadcount<-mean(samples$Freq)
platewide_sd_beadcount<-sd(samples$Freq)
pdf(file.path(outpath,"000_average_beadcounts.pdf"))
plot(samples$Freq,pch=46,col="red",cex=5,ylim=c(0,(platewide_average_beadcount+(6*platewide_sd_b
eadcount))),ylab="average droplet count and 99% CI",xaxt="n")
abline(h=(platewide_average_beadcount+2.59*platewide_sd_beadcount), col="PINK", lty=1)
abline(h=(platewide_average_beadcount-2.59*platewide_sd_beadcount), col="PINK", lty=1)
axis(1,rownumbers,labels=samples$Var1,cex.axis=0.4,las=2)
grid(nx=100,ny=NULL)
dev.off()
#QC warning about bead count
samples$dropletcountwarning<-NA
for (i in rownumbers) {if
(samples$Freq[i]>platewide_average_beadcount+(2.59*platewide_sd_beadcount)){samples$dropletcount
warning[i]<-"Droplet count value is greater than 99% upper CI limit for the plate"}}
```

```
for (i in rownumbers) {if (samples$Freq[i]<platewide_average_beadcount-
(2.59*platewide_sd_beadcount)){samples$dropletcountwarning[i]<-"Droplet count value is lesser
than 99% upper CI limit for the plate"}}
for (i in rownumbers) {if
(samples$Freq[i]>platewide_average_beadcount+(2.59*platewide_sd_beadcount)){samples$FAILS[i]<-
"Droplet count value is greater than 99% upper CI limit for the plate"}}
for (i in rownumbers) {if (samples$Freq[i]<platewide_average_beadcount-
(2.59*platewide_sd_beadcount)){samples$FAILS[i]<-"Droplet count value is lesser than 99% upper
CI limit for the plate"}}
for (i in rownumbers) {if (samples$Freq[i]<10000){samples$FAILS[i]<-"Droplet count is below
10000"}}

rm(current_na)

current_na<-is.na(samples$Freq)
for (i in rownumbers) {if (current_na[i]==TRUE){samples$dropletcountwarning[i]<-"No droplets"}}
for (i in rownumbers) {if (current_na[i]==TRUE){samples$FAILS[i]<-"No droplets"}}


#calculate values for poisson calculation.
#phat is estimator of P, the probability of a micelle being positive. P is unknown but phat can
be estimated by phat = number of positive droplets / total droplets
samples$phat_fam<-((samples$famposvicneg+samples$famposvicpos)/samples$Freq)
samples$phat_vic<-((samples$famnegvicpos+samples$famposvicpos)/samples$Freq)

#standard deviation of p_hat estimator
samples$sd_phat_fam<- sqrt((samples$phat_fam*(1-samples$phat_fam))/samples$Freq)
samples$sd_phat_vic<- sqrt((samples$phat_vic*(1-samples$phat_vic))/samples$Freq)

#upper and lower confidence intervals (95%) of p_hat estimates
samples$phat_low_fam <- samples$phat_fam - (1.96 * samples$sd_phat_fam)
```

```
samples$phat_high_fam <- samples$phat_fam + (1.96 * samples$sd_phat_fam)

samples$phat_low_vic <- samples$phat_vic - (1.96 * samples$sd_phat_vic)
samples$phat_high_vic <- samples$phat_vic + (1.96 * samples$sd_phat_vic)

#lambda is the true concentration of target molecules per chamber. It is unknown but can be
estimated by lambda hat. lambda_hat =  -ln (1-p_hat)

samples$lambda_hat_fam<-(-log(1-samples$phat_fam))
samples$lambda_hat_vic<-(-log(1-samples$phat_vic))

#upper and lower confidence intervals for lambda hat :
samples$lambda_hat_low_fam <- (-log(1-samples$phat_low_fam))
samples$lambda_hat_high_fam <- (-log(1-samples$phat_high_fam))

samples$lambda_hat_low_vic <- (-log(1-samples$phat_low_vic))
samples$lambda_hat_high_vic <- (-log(1-samples$phat_high_vic))

# lambda_hat = concentration target per micelle
# lambda_hat_low = lower 95% CI for lambda hat
# lambda_hat_high = upper 95% CI for lambda hat

# calculate concentration of target molecules per reaction.

samples$fam_copies_per_reaction<-samples$lambda_hat_fam*samples$Freq
samples$vic_copies_per_reaction<-samples$lambda_hat_vic*samples$Freq

samples$fam_copies_per_reaction_low<-samples$lambda_hat_low_fam*samples$Freq
samples$fam_copies_per_reaction_high<-samples$lambda_hat_high_fam*samples$Freq
```

```
samples$vic_copies_per_reaction_low<-samples$lambda_hat_low_vic*samples$Freq
samples$vic_copies_per_reaction_high<-samples$lambda_hat_high_vic*samples$Freq



samples$volume<-samples$Freq*0.91e-3
samples$concentration_fam_average_copies_uL <- samples$fam_copies_per_reaction/samples$volume
samples$concentration_fam_low_copies_uL <- samples$fam_copies_per_reaction_low/samples$volume
samples$concentration_fam_high_copies_uL <- samples$fam_copies_per_reaction_high/samples$volume

samples$concentration_vic_average_copies_uL <- samples$vic_copies_per_reaction/samples$volume
samples$concentration_vic_low_copies_uL <- samples$vic_copies_per_reaction_low/samples$volume
samples$concentration_vic_high_copies_uL <- samples$vic_copies_per_reaction_high/samples$volume

for (i in
rownumbers){if(is.na(samples$concentration_fam_high_copies_uL[i])){samples$concentration_fam_hig
h_copies_uL[i]<-samples$concentration_fam_average_copies_uL[i]}else{next}}
for (i in
rownumbers){if(is.na(samples$concentration_vic_high_copies_uL[i])){samples$concentration_vic_hig
h_copies_uL[i]<-samples$concentration_vic_average_copies_uL[i]}else{next}}

for (i in
rownumbers){if(is.infinite(samples$concentration_fam_average_copies_uL[i])){samples$concentratio
n_fam_average_copies_uL[i]<-20000}}
for (i in
rownumbers){if(is.infinite(samples$concentration_fam_low_copies_uL[i])){samples$concentration_fa
m_low_copies_uL[i]<-20000}}
for (i in
rownumbers){if(is.infinite(samples$concentration_fam_high_copies_uL[i])){samples$concentration_f
am_high_copies_uL[i]<-20000}}
```

```
for(i in
rownumbers){if(is.infinite(samples$concentration_vic_average_copies_uL[i])){samples$concentratio
n_vic_average_copies_uL[i]<-20000}}
for(i in
rownumbers){if(is.infinite(samples$concentration_vic_low_copies_uL[i])){samples$concentration_vi
c_low_copies_uL[i]<-20000}}
for(i in
rownumbers){if(is.infinite(samples$concentration_vic_high_copies_uL[i])){samples$concentration_v
ic_high_copies_uL[i]<-20000}}



#plot graphs of calculated concenentration (copies/uL) values with error bars
#library(gplots)

pdf(file.path(outpath,"001_concentration_vic.pdf"))
plot
(samples$concentration_vic_average_copies_uL,pch=46,cex=4,log="y",xlab="Sample",ylab="HURNASE
(copies/uL)",xaxt="n",cex.axis=0.4,xlim=c(0,100),ylim=c(0.1,(max(samples$concentration_vic_high_
copies_uL)*1.1)))
for(i in
rownumbers){arrows(i,samples$concentration_vic_average_copies_uL[i],i,samples$concentration_vic_
high_copies_uL[i],length=0.02,angle=90,code=2,col="red")}
for(i in
rownumbers){arrows(i,samples$concentration_vic_average_copies_uL[i],i,samples$concentration_vic_
low_copies_uL[i],length=0.02,angle=90,code=2,col="red")}
axis(1,rownumbers,labels=samples$Var1,cex.axis=0.4)
grid()
dev.off()
```

```
pdf(file.path(outpath,"001_concentration_fam.pdf"))
plot
(samples$concentration_fam_average_copies_uL,pch=46,cex=4,log="y",xlab="Sample",ylab="HURNASE
(copies/uL)",xaxt="n",cex.axis=0.4,xlim=c(0,100),ylim=c(0.1,(max(samples$concentration_fam_high_
copies_uL)*1.1)))
for(i in
rownumbers){arrows(i,samples$concentration_fam_average_copies_uL[i],i,samples$concentration_fam_
high_copies_uL[i],length=0.02,angle=90,code=2,col="red")}
for(i in
rownumbers){arrows(i,samples$concentration_fam_average_copies_uL[i],i,samples$concentration_fam_
low_copies_uL[i],length=0.02,angle=90,code=2,col="red")}
axis(1,rownumbers,labels=samples$Var1,cex.axis=0.4)
grid()
dev.off()



#DEFINE FAILS AND RESULTS
for (i in rownumbers) {if (samples$concentration_fam_low_copies_uL[i]<0){samples$FAILS[i]<-
"WARNING : CI for FAM crosses zero"}}
for (i in rownumbers) {if (samples$famnegvicneg[i]<(0.01*samples$Freq[i])){samples$FAILS[i]<-
"WARNING: REACTION MAY BE SATURATED WITH TOO MANY TEMPLATES"}}
for (i in rownumbers) {if
(samples$concentration_vic_low_copies_uL[i]<0.0000001){samples$FAILS[i]<-"FAIL: HUMAN DNA
CONCENTRATION TOO LOW"}}

#define amount of area under the curve that is above zero
for (i in rownumbers){samples$areaabovezerofam[i]<-1-
pnorm(0,mean=samples$phat_fam[i],sd=samples$sd_phat_fam[i])}
```

```
for (i in rownumbers){samples$areaabovezerovic[i]<-1-
pnorm(0,mean=samples$phat_vic[i],sd=samples$sd_phat_vic[i])}

#assign endogenous control status
samples$endogenouscontrolresult<-NA
for (i in rownumbers) {if
(samples$areaabovezerovic[i]>=0.95){samples$endogenouscontrolresult[i]<-"Endogenous control
OK"}}
for (i in rownumbers) {if
(samples$areaabovezerovic[i]<0.90){samples$endogenouscontrolresult[i]<-"Endogenous control
failed"}}

#assign warnings based on FI average values and 95% CI platewide
samples$targetresult<-NA
for (i in rownumbers) {if (samples$areaabovezerofam[i]>=0.95){samples$targetresult[i]<-"Sample
is positive for the target"}}
for (i in rownumbers) {if (samples$areaabovezerofam[i]<0.90){samples$targetresult[i]<-"Sample is
negative for the target"}}


#print output
write.table(samples, file =
(file.path(outpath,"000_results_full.txt")),row.names=F,col.names=T,quote=F,sep="\t")
columns<-c(1,55,56,47,48,49,50,51,52,53,54,2,13,14,15,16,11,12,46)
write.table(samples[,columns], file =
(file.path(outpath,"000_results_concise.txt")),row.names=F,col.names=T,quote=F,sep="\t")

##########################################################################################
#END OF SCRIPT dropletR_man_thresholds.R
##########################################################################################
```
